# Rapid and Automated Approach for Early Crop Mapping Using Sentinel-1 and Sentinel-2 on Google Earth Engine; A Case of a Highly Heterogeneous and Fragmented Agricultural Region

**DOI:** 10.3390/jimaging8120316

**Published:** 2022-11-24

**Authors:** Hajar Saad El Imanni, Abderrazak El Harti, Mohammed Hssaisoune, Andrés Velastegui-Montoya, Amine Elbouzidi, Mohamed Addi, Lahcen El Iysaouy, Jaouad El Hachimi

**Affiliations:** 1 Geomatics, Georesources and Environment Laboratory, Faculty of Sciences and Techniques, Sultan Moulay Slimane University, Beni Mellal 23023, Morocco; 2 Applied Geology and Geo-Environment Laboratory, Faculty of Sciences, Ibn Zohr University, Agadir 80000, Morocco; 3 Faculty of Applied Sciences, Ibn Zohr University, Ait Melloul 86150, Morocco; 4 Centro de Investigación y Proyectos Aplicados a las Ciencias de la Tierra (CIPAT), ESPOL Polytechnic University, Guayaquil P.O. Box 09-01-5863, Ecuador; 5 Facultad de Ingeniería en Ciencias de la Tierra (FICT), ESPOL Polytechnic University, Guayaquil P.O. Box 09-01-5863, Ecuador; 6 Geoscience Institute, Federal University of Pará, Belém 66075-110, Brazil; 7 Laboratoire d’Amélioration des Productions Agricoles, Biotechnologie et Environnement (LAPABE), Faculté des Sciences, Université Mohammed Premier, Oujda 60000, Morocco; 8 ERSC, LEC, Research Center E3S, EMI, Mohammed V University in Rabat, BP765 Agdal, Rabat 10106, Morocco

**Keywords:** crop type mapping, Sentinel-1, Sentinel-2, machine learning, Google Earth Engine, time-series

## Abstract

Accurate and rapid crop type mapping is critical for agricultural sustainability. The growing trend of cloud-based geospatial platforms provides rapid processing tools and cloud storage for remote sensing data. In particular, a variety of remote sensing applications have made use of publicly accessible data from the Sentinel missions of the European Space Agency (ESA). However, few studies have employed these data to evaluate the effectiveness of Sentinel-1, and Sentinel-2 spectral bands and Machine Learning (ML) techniques in challenging highly heterogeneous and fragmented agricultural landscapes using the Google Earth Engine (GEE) cloud computing platform. This work aims to map, accurately and early, the crop types in a highly heterogeneous and fragmented agricultural region of the Tadla Irrigated Perimeter (TIP) as a case study using the high spatiotemporal resolution of Sentinel-1, Sentinel-2, and a Random Forest (RF) classifier implemented on GEE. More specifically, five experiments were performed to assess the optical band reflectance values, vegetation indices, and SAR backscattering coefficients on the accuracy of crop classification. Besides, two scenarios were used to assess the monthly temporal windows on classification accuracy. The findings of this study show that the fusion of Sentinel-1 and Sentinel-2 data can accurately produce the early crop mapping of the studied area with an Overall Accuracy (OA) reaching 95.02%. The scenarios prove that the monthly time series perform better in terms of classification accuracy than single monthly windows images. Red-edge and shortwave infrared bands can improve the accuracy of crop classification by 1.72% when compared to only using traditional bands (i.e., visible and near-infrared bands). The inclusion of two common vegetation indices (The Normalized Vegetation Index (NDVI), the Enhanced Vegetation Index (EVI)) and Sentinel-1 backscattering coefficients to the crop classification enhanced the overall classification accuracy by 0.02% and 2.94%, respectively, compared to using the Sentinel-2 reflectance bands alone. The monthly windows analysis indicated that the improvement in the accuracy of crop classification is the greatest when the March images are accessible, with an OA higher than 80%.

## 1. Introduction

Morocco’s climate is classified as semi-arid to arid, and it is considered one of the countries most affected by climate change [1,2]. The Tadla Irrigated Perimeter (TIP) is one of the most heterogeneous and fragmented agricultural regions in Morocco. It contributes to the national production of sugar beet, cereals, olive, citrus, and pomegranate. The TIP, as well as the other Moroccan irrigated perimeters, are expected to develop irrigation management techniques to increase production and save water [3].

Recently TIP studies focused on mapping crop types using Landsat 8 NDVI data [4] and classifying crops using the Sentinel-2 time series [5]. The Tadla Irrigated Perimeter shows potential for improving the quality of early crop identification through the evaluation of different sensors. To efficiently handle the challenges of big data processing, Google has created a cloud computing platform called Google Earth Engine [6].

A more accurate crop classification may therefore result from the new Google Earth Engine cloud platform’s effectiveness in remote sensing accessibility, reduction of processing time, computation, and automation.

Therefore, the present work is the first study in this region that proposes a cloud computing approach developed in the GEE JavaScript interface to evaluate the different band combinations of the high spatiotemporal resolution of Sentinel-1 and Sentinel-2 for early-season crop mapping.

Remote sensing is increasingly used for recognizing crop condition information. Data availability has improved, as well as spatial, temporal, and spectral resolutions. [7]. Despite the fact that large-scale agriculture is a global trend for agricultural development, family farming continues to be the primary management form of agricultural practices in developing countries, namely Morocco [8,9] This type of farming is characterized by small plots, making the distinction and classification of crops a difficult issue. As in the TIP region, the size of the plots varies from 0.5 to 10 hectares; 86% of them have an area of less than 5 ha, 8% range from 5 to 10 ha, and only 5% have an area greater than 10 ha.

Moderate Resolution Imaging Spectroradiometer (MODIS) images at 500 m resolution are mostly used in large-scale crop mapping. However, to categorize regions with small plots, a medium to high resolution is required. [10]. Several studies have been conducted to map small-scale agriculture [11,12,13].

The recent cloud-based platform GEE provides open access remote sensing datasets and offers a new choice for researchers focused on geospatial capabilities, with planetary-scale analysis [14,15]. GEE has recently gained many remote sensing applications, such as cropland mapping. For example, Xiong et al. [13] developed an automatic algorithm within GEE to classify croplands over the entire African continent, Shelestov et al. [14] conducted a crop classification analysis using huge amounts of multi-temporal data and GEE, and Kolli et al. [15] assessed the change in the extent of mangrove ecosystems using different spectral indices and random forests in GEE. Moreover, Amani et al. [16] used the GEE cloud computing platform with an Artificial Neural Network (ANN) algorithm to produce an object-based map.

One of the most intriguing sources of crop information is optical satellite imagery. However, it has limitations and drawbacks during cloudy periods [17]. This challenge causes great difficulties in identifying crops, setting up monitoring practices, and managing irrigation water. One potential remedy for this problem is SAR remote sensing. SAR is an active technique that provides cloud-free imagery, both during the day and at night, and in all weather conditions [11]. Furthermore, SAR sensors are sensitive to the physical and dielectric properties that also include the morphology of crops, providing more details about the type of vegetation cover [17].

The fusion of Optical and SAR data is a powerful tool for developing classification procedures [18]. Several studies have shown that crop classification accuracy improves when optical and SAR data are combined [18,19].

The use of multi-temporal remote sensing data for improved spectral feature recognition and change detection is key to crop-type mapping [20]. Many studies have highlighted the performance of time series imagery in the classification process. For example, Inglada et al. [19] found that using SAR and optical image time series improved early crop type identification, and Van Tricht et al. [17] concluded that synergistic use of radar Sentinel-1 and optical Sentinel-2 multi-temporal imagery provides more precise information and an improvement in classification accuracy.

A variety of techniques for balancing and regenerating dense time series have been proposed. Equal interval synthesis, generating median composite images, is a widely accepted pixel synthesis method for classification tasks and other works [10].

Crop mapping from satellite images has been performed using many classification methods. In recent years, machine learning algorithms have appeared as more accurate alternatives, particularly for large dimensional and complex data [20] (Table 1). The Random Forest (RF) algorithm has been used in several studies related to crop classification, which has demonstrated good performance [21]. Additionally, the RF algorithm has produced reliable results in numerous investigations that predicted soil properties using regression models [22].

The selection of appropriate feature exploration derived from satellite imagery is critical for accurate crop type classification. In South China, the selection of Sentinel-1 and Sentinel-2 features was crucial for the discrimination between early, middle, and late rice [23].

According to some recent studies, the red-edge bands of Sentinel-2 were useful for estimating crop production [24] and changes in vegetative moisture content [25], while the shortwave infrared (SWIR) bands were found to be suitable for analyzing vegetation stress [26].

Therefore, this research aims to highlight the effect of red edge, shortwave infrared (SWIR) bands, and vegetation indices on classification accuracy.

Determining the most appropriate time window for accurate discrimination of crops is key for timely agricultural policy decisions; in Heilongjiang Province, China, LUO et al. [10] revealed the earliest accurate timing for crop classification of soybean, rice, and corn by performing a combination of Landsat 8 OLI and Sentinel-2 data with the reference time series-based method (RBM).

Thus, the present study analyzes the change in accuracy parameters during the growing season to deduce the optimal time window for an accurate TIP crop classification.

Furthermore, early crop mapping has the benefit of improving decision-making in agricultural practices, yield increases, and water resource management. In Italy, Azar et al. [27] used spectral indices derived from multi-temporal Landsat 8 OLI images and supervised classification algorithms to evaluate the performance of early-season crop classification.

As a result, the TIP, as well as the other Moroccan irrigated perimeters, are expected to be able to deduce the optimal time window for the discrimination of crops during the early growing season. Early crop classification results would be beneficial for enhancing decision-making about agricultural practices, production improvements, and water resource management. Additionally, the use of cloud computing would significantly improve access to remote sensing data and save time.

The research objectives of this paper are:

(1) To evaluate the high spatiotemporal resolution of Sentinel-1, Sentinel-2, and RF machine learning algorithms for accurate and early crop type mapping in a heterogeneous and fragmented agricultural region on the GEE platform by analysing the contribution of the various bands in improving the classification accuracy.
(2) To assess the individual monthly temporal windows and the entire monthly time series on classification accuracy.

**Table 1:** Recent studies in crop classification techniques using Sentinel-1 and Sentinel-2.

Author	Year	Problem Definition	Targeted Crop	Dataset	Model	Accuracy
Tufail et al. [28]	2022	Crop type mapping	Wheat, strawberry, fodder, and rice	Sentinel-1, Sentinel-2	RF	97%
He et al. [23]	2021		Rice	Sentinel-1, Sentinel-2	RF	81%
Rao et al. [29]	2021		Maize, mustard, tobacco, and wheat)	Sentinel-1, Sentinel-2, and PlanetScope	SVM	85%
Schulz et al. [30]	2021		Rice, cropland, and sparse vegetation	Sentinel-1, Sentinel-2	RF	73.3%
					SVM	60.8%
					ML	31.7%

## 2. Study Area

**Figure 1:**
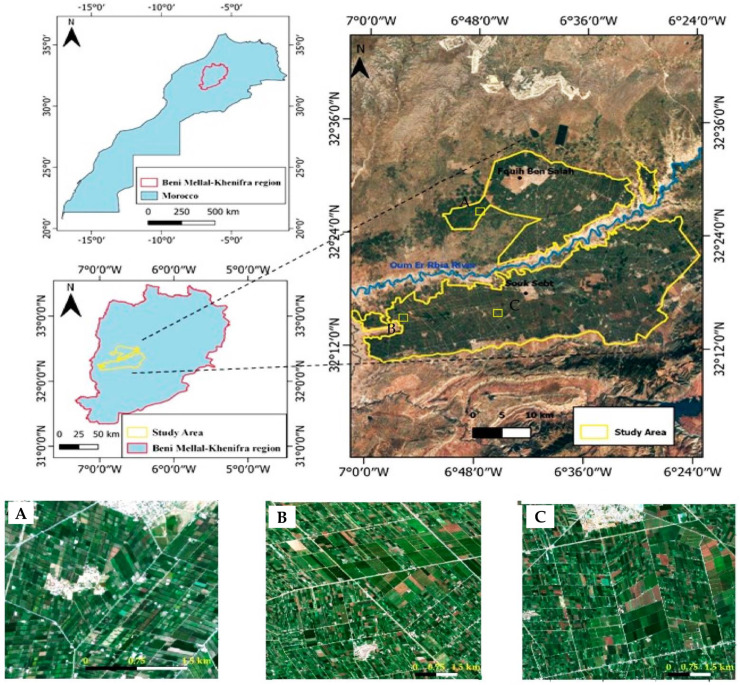
Location of the study area at the national scale (**left**). Scene of Tadla Irrigated Perimeter (**right**). (**A**–**C**) a detailed zoom of the study area Sentinel-2A image in true color.

The Tadla Irrigated Perimeter is an irrigated system situated in central Morocco between 32°12′0″ N 7°0′0″ W and 32°24′0″ N 6°24′0″ W, with an average altitude of 400 m (Figure 1), including more than 100 000 ha of irrigated land. The TIP is subdivided into two compartments separated by the Oum Er Rbia river: the perimeter of Beni Amir on the northern bank of the river, supplied by the Chahid Ahmed El Hansali Dam with a capacity of 670 million m^3^, and the perimeter of Beni Moussa in the southern bank, supplied by the Bin El Ouidane Dam, with a capacity of 1.3 billion m^3^. This water supply is the source of the diversity of crops in the region. The most dominant crops in the region are cereals (wheat and barley), alfalfa, sugar beet, citrus, olives, and pomegranate. The area is characterized by a very fragmented and intensive agricultural system; 86% of parcels are less than 5 ha, and only 5% of the parcels exceed 10 ha [4]. The climate is arid to semi-arid, with a temperature that varies between 6° (January) and 48° (August). The average annual precipitation is 280 mm, with important annual variations [31].

## 3. Materials and Methods

### 3.1. Ground Data

**Figure 2:**
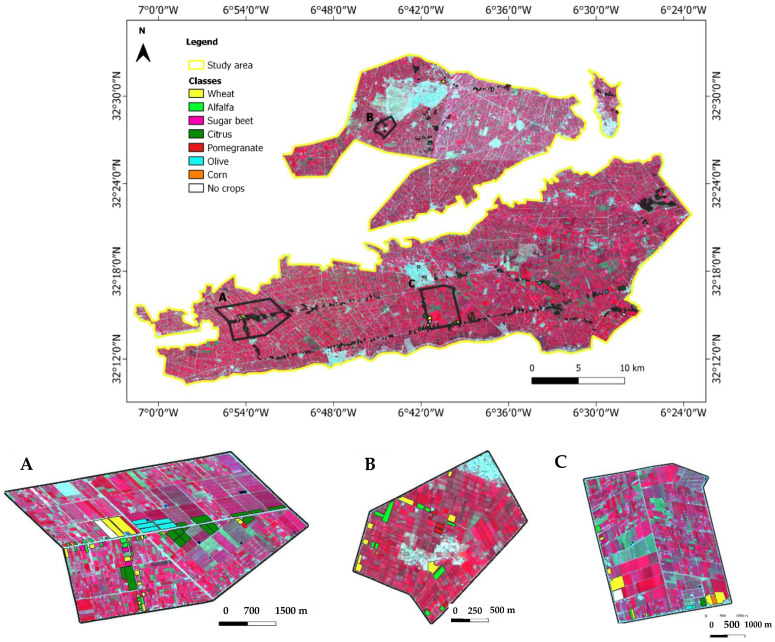
Spatial distribution of the ROIs and the corresponding Sentinel-2 image (False Color Combination: NIR, Red, Green band as RGB), (**A**–**C**) a detailed zoom.

The TIP crops were identified throughout field campaigns from September 2020 to March 2021 as Regions of Interest (ROIs). They were chosen in various locations and parcel sizes. Figure 2 depicts the spatial distribution of the ROIs, while Table 2 presents the characteristics of selected ground data. The coordinates of the ROIs were recorded using a GPS and the QField application. QField is dedicated to the easy and efficient execution of GIS fieldwork and the comfortable exchange of data between the field and the office.

**Figure 3:**
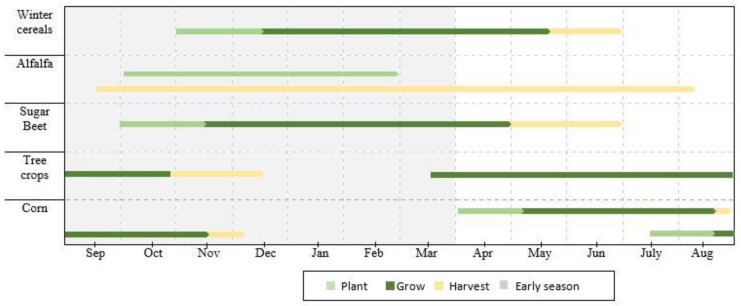
Calendar of planting, growing, and harvesting dates of the TIP crops for the growing season 2020/2021.

The major crops identified were winter cereals (wheat and barley), alfalfa, sugar beet, corn, citrus, pomegranate, and olive. These data were used for classifying and assessing the accuracy of the produced maps. The number of ROIs collected was 736 plots, including 512 for training (70%) and 224 for validation (30%). Figure 3 shows the phenological stages of growth for the major crops in the TIP.

### 3.2. Satellite Data

#### 3.2.1. Sentinel-2

Two satellites in orbit, Sentinel-2A and Sentinel-2B, are equipped with a Multi-spectral instrument (MSI), allowing the observation of the Earth at spatial resolutions of 10 m, 20 m, and 60 m [32]. The Level-2A product provides Bottom Of Atmosphere (BOA) reflectance images. The MSI has 13 spectral bands, of which ten bands are from visual to shortwave infrared, including four bands with a 10-m spatial resolution (blue, green, red, near-infrared) and six bands with 20-m resolution (red-edge 1, red-edge 2, red-edge 3, red-edge 4, SWIR1, and SWIR2). The selection of satellite images was based on the criteria that the scenes should have minimal or no clouds (less than 2% cloud cover), with a sufficient number of days for each month. In this study, 38 images were considered (Table 3).

#### 3.2.2. Sentinel-1

Sentinel-1 is a system of two polar-orbiting satellites (Sentinel-1 A/B) that are active day and night. This study considered the C-band synthetic aperture radar, the interferometric wide swath (IWS) mode in the ascending view angle at the ground range detected (GRD) product level-1. The vertical transmit/receive (VV), and the horizontal/vertical transmit/receive (VH) were processed in this research. The number of Sentinel-1 images processed was 45. (Table 3). However, on 3 August 2022, the European Space Agency and the European Commission announced the end of the Copernicus Sentinel-1B satellite mission.

### 3.3. Tools Used

The Google Earth Engine (GEE) environment was used to develop the workflow to extract band reflectance values, vegetation indices, and backscattering coefficients from Sentinel-2 and Sentinel-1 time series images. The crop classification results were exported as Geocoded rasters into QGIS for visual interpretation.

## 4. Methodology

This paper serves to map, accurately and early, the crop types in a highly heterogeneous and fragmented landscape region using the high spatiotemporal resolution of Sentinel-1 and Sentinel-2 by performing an RF classifier on GEE. In this context, five experiments were performed to examine the band reflectance values, vegetation indices, and backscattering coefficients on crop classification. Additionally, two temporal scenarios for crop classification were evaluated.

**Figure 4:**
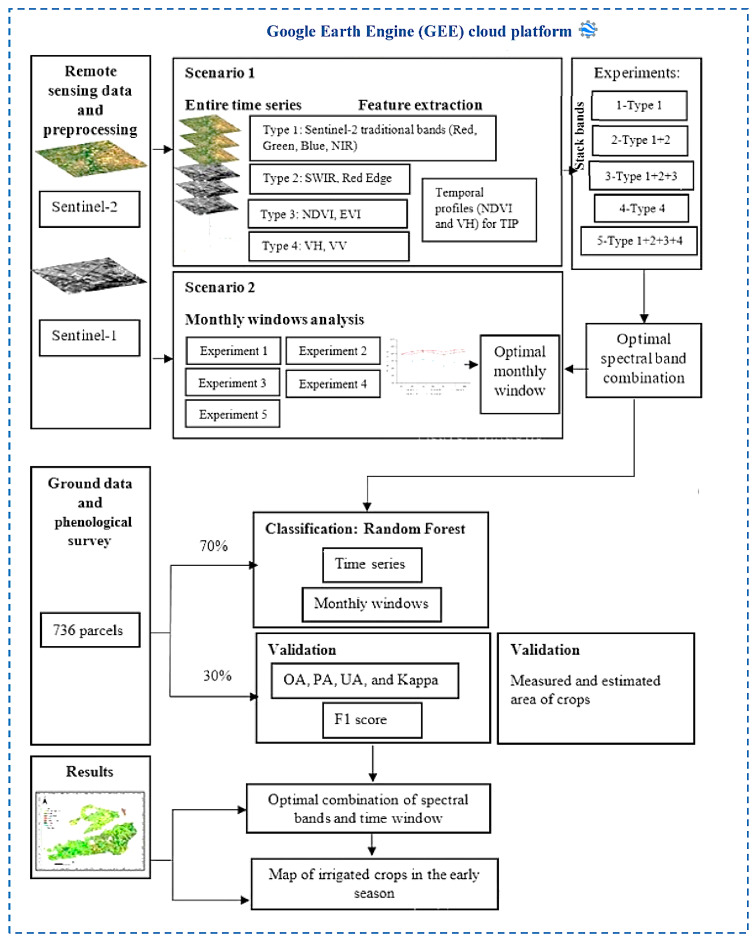
Flowchart of the proposed method for producing crop mapping using machine learning based on five experiments and two scenarios.

The workflow of the proposed methodology is shown in Figure 4.

### 4.1. Pre-Processing

In this section, Sentinel-2 was selected to classify both 10- and 20-m resolution bands. The bands with 20 m resolution were resampled to 10 m using the nearest-neighbour interpolation.

The Normalized Vegetation Index (NDVI) [33] and the Enhanced Vegetation Index (EVI) [34] were added as inputs for the classification.

The NDVI can be considered one of the most important vegetation indices used for identifying the growing conditions of crops [19]. It is obtained from reflectances in the red (R) and near-infrared (NIR) portions of the spectrum [35] (Table 4).

The Enhanced Vegetation Index (EVI), which is described as ‘an optimized vegetation index to deliver an accurate vegetation signal with increased sensitivity in lands with dense biomass’, has received much interest in monitoring the quality and amount of vegetation [36]. EVI is derived from reflectances in the red (R), blue (B), and near-infrared (NIR) portions of the spectrum [35] (Table 4).

The backscattering coefficient, provided by the Sentinel-1 toolbox, offers additional information on crop mapping [9]. The backscattering time series with VV and VH polarization were combined with Sentinel-2 data for the classification process. The GEE supplies pre-processed Sentinel-1 data [37].

### 4.2. Image Compositing

**Figure 5:**
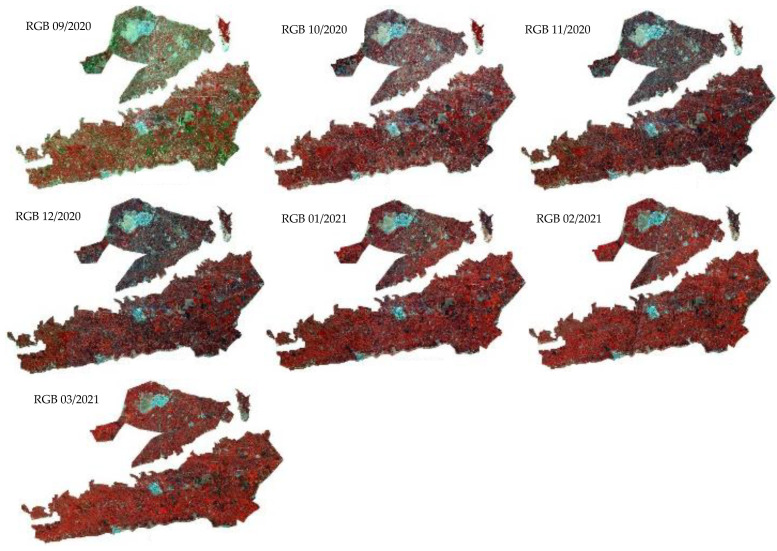
Sentinel-2 RGB monthly images (False Color Combination: NIR, Red, Green band as RGB) from September 2020 to March 2021 of the Tadla Irrigated Perimeter (TIP).

**Figure 6:**
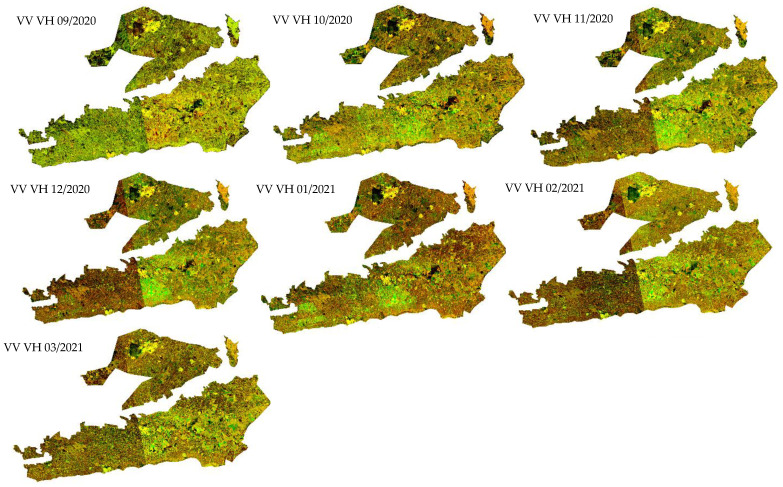
Backscattering monthly images from September 2020 to March 2021 of the Tadla Irrigated Perimeter (TIP).

The time series data provide additional specific information about spectral features and change detection [20]. The statistical information about multi-temporal data has successfully demonstrated their performance in crop classification and differentiation of land types [10]. Furthermore, the median time series is a robust statistical indicator in crop classification. Consequently, the monthly median was selected as a statistical parameter of the time series images. The median composite images of Sentinel-2 reflectance bands, vegetation indices, and Sentinel-1 backscattering coefficients at VH and VV polarization were generated for each month from September to March in the TIP region during the early growing season (Figure 5 and Figure 6).

### 4.3. Scenario 1

In this scenario, the GEE JavaScript interface was performed to select the entire time series of Sentinel-1 and Sentinel-2 monthly composite images, covering the period from 2 September 2020, to 24 March 2021. To evaluate the different band reflectance values, vegetation indices, and backscattering coefficients on crop classification, we designed the following experiments.

*Experiment 1:* Only Sentinel-2 traditional bands (visible bands and NIR bands) were used for classification.

*Experiment 2:* The traditional bands, SWIR, and red-edge bands were used for classification.

*Experiment 3:* The traditional bands, SWIR bands, red-edge bands, and vegetation indices (NDVI and EVI) were used for classification.

*Experiment 4:* Only the SAR (Sentinel-1) data were used for classification.

*Experiment 5:* All the optical (Sentinel-2) and SAR (Sentinel-1) data were used for classification.

Sentinel-1 and Sentinel-2 bands were stacked to conduct these experiments.

### 4.4. Scenario 2

To investigate the effectiveness and the accuracy of classification through the monthly windows, the analysis was executed under the aforementioned five different experiments. For this purpose, another JavaScript interface was developed to produce 7 crop classification maps ranging from September to March under the five experiments aiming to deduce both optimal experiments and monthly windows.

### 4.5. Classification Process

Many crop classification investigations have confirmed the usefulness of RF in crop recognition [28]. The RF algorithm can be described as a collection of various decision trees, where each tree provides one vote for the most prevalent class [38]. It is a robust classifier that solves the overfitting problem associated with Decision Tree (DT) classifiers by constructing a set of DT classifiers [39]. This study used the performance of the RF classifier included in GEE to classify the TIP crops at an early season under two scenarios and five experiments.

### 4.6. Validation

Five confusion metrics, including overall accuracy (OA), Kappa coefficient, user accuracy (UA), producer accuracy (PA), and F1 score, were used to evaluate the crop classification result. For training purposes, 70% of each class’s ROIs were randomly chosen, while the additional 30% of field survey sites were employed to examine the accuracy. The OA was computed by summing the number of successfully classified cells and dividing by the total number of cells, while the Kappa coefficient reflects the agreement between classification and truth values [40].

The PA represents the conditional probability that a specific location on the classification map’s output is consistent with any random sample in the test data, while the UA consists of selecting a random sample with the same conditional probability as the actual type of ground from the classification results [10]. The PA and UA were generated from the error matrix of classification.

The F1 score is a critical metric indicator that optimizes the dispersion between PA and UA for each class by the generation of the harmonic mean of PA and UA [41]. The following equations were used to calculate these metrics:

(1) $$OA\left(\%\right)=\frac{\sum_{i=1}^{n}p_{ii}}{N}\times 100$$

(2) $$Kappa=\frac{N\sum_{i=1}^{n}p_{ii}-\sum_{i=1}^{n}(p_{i+}\times p_{+i})}{N^{2}-\sum_{i=1}^{n}(p_{i+}\times p_{+i})}$$

(3) $$UA\left(\%\right)=\frac{p_{ii}}{p_{i+}}\times 100$$

(4) $$PA\left(\%\right)=\frac{p_{ii}}{p_{+i}}\times 100$$

(5) $$F1\;score\left(\%\right)=\frac{UA\times PA}{UA+PA\;}\times 2$$

here *n* is the total number of columns of the confusion matrix; $p_{ii}$ is the number of correctly classified upper crop type samples in the *i* row and *i* column of the confusion matrix, $p_{i+}$ and $p_{+i}$ are the total number of crop-type samples in row *i* and column *i*, and *N* is the total number of samples included for verification.

## 5. Results

### 5.1. Temporal Profiles of Normalized Difference Vegetation Index (NDVI)

**Figure 7:**
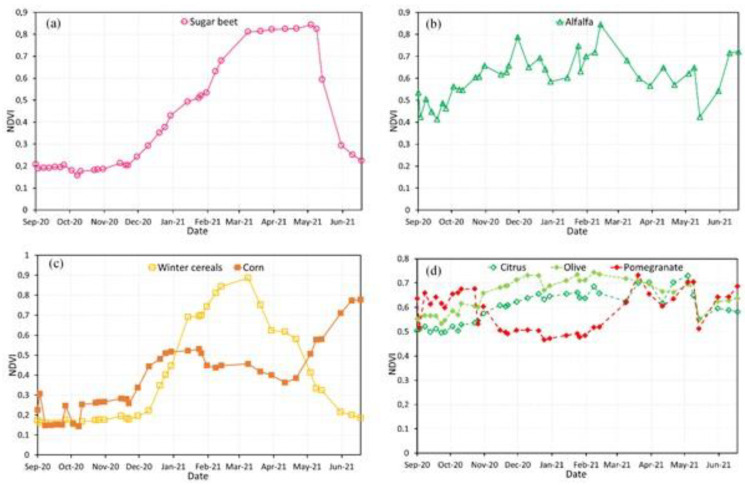
NDVI spectral curves for major crop types planted in t2020/2021: (**a**) Sugar beet; (**b**) Alfalfa; (**c**) Winter cereals (wheat and barley) and corn; (**d**) Tree crops (pomegranates, citrus, and olive).

The main crops in the TIP are winter cereals, sugar beet, alfalfa, corn, and tree crops such as citrus, pomegranate, and olive. This section focused on monitoring the phenological development of TIP crops by analyzing the NDVI time series profiles. These profiles include the average NDVI of the reference parcels on each date (Figure 7).

For sugar beet, sowing was undertaken from the end of September to the beginning of November, after rainy periods, ensuring enough soil moisture for germination. From this period, the vegetation indices values increase until reaching maximum values between March and May (NDVI > 0.8), while the NDVI values decrease in late May, allowing harvesting to begin.

Alfalfa is a forage crop with high productivity, long duration (3–4 years), and the ability to regrow [4]. It is commonly planted from October to February. As a result, the NDVI values of this crop show a rapid change that is replicated throughout its growth cycle due to the cycling between harvest and regrowth.

Cereal grains include winter cereals (wheat and barley) and corn. They were examined separately due to the differences in developmental stages. For winter cereals, sowing took place from late October to December, increasing the NDVI values until late March (NDVI > 0.8). The decrease in the NDVI values for winter cereals occurs before sugar beet. Harvesting can be carried out from late May to early June.

Corn can be planted in two seasons, including March to April and July to August, while harvesting happens from late July to early August in the first season and from late November to early December in the second season. As shown in Figure 7, sowing took place in the first season of planting corn, showing an increase in the NDVI values from March to April.

For tree crops (citrus, pomegranate, and olive), the NDVI values are greater than 0.4 throughout the season. The elevated NDVI values are attributed to the high rate of chlorophyll along the phenological stages of development. However, slight decreases in NDVI values are observed during the period between November to January since this period is known for leaf loss (e.g., pomegranate) or farming practice (e.g., cutting citrus), while olive trees keep the same rate of greenness, which results in more constant NDVI values.

### 5.2. Temporal Profiles of Backscattering in VH Polarization

**Figure 8:**
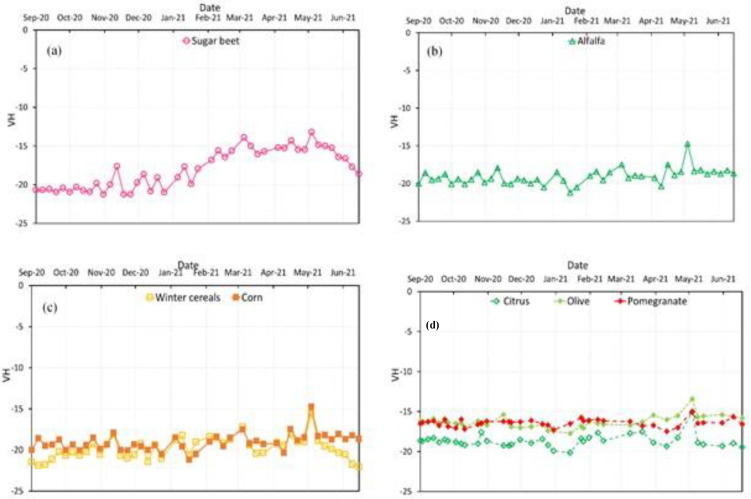
Backscattering spectral curves for major crop types planted in 2020/2021: (**a**) Sugar beet; (**b**) Alfalfa; (**c**) Winter cereals (wheat and barley) and corn; (**d**) Tree crops (pomegranates, citrus, and olive).

In this section, the profiles show the average SAR backscatter (σ ◦) in the VH polarization of the reference plots at each date (Figure 8).

For sugar beet, the period of sowing, growing, and harvesting was recorded according to the NDVI profile, with an increase in backscatter values during the growth phase of the plant, reaching maximum values during the period between March and May. In late May, the backscatter (σ ◦) in VH polarization values tend to decline, permitting harvesting to begin.

Alfalfa shows continuous variation throughout the season, with backscatter coefficient values ranging from −20 to −15. The phenological stages of winter cereals, corn, and tree crops were less differentiated for the backscatter coefficient in VH polarization.

### 5.3. Crop Mapping in the Early Season with the Entire Time Series (Scenario 1)

The TIP crop mapping developed with the entire time series was based on pixel-based image classification of monthly median images from optical and SAR data from September 2020 to March 2021, using five experiments.

**Figure 9:**
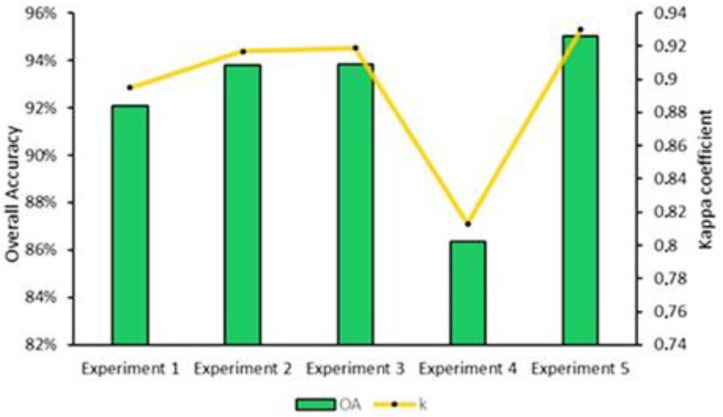
Classification accuracy of experiments using time series images.

As shown in Figure 9, the classification accuracy of experiment 2 (93.80% and 0.92) is higher than experiment 1 (92.08% and 0.89), which demonstrates that the addition of the SWIR bands and the red-edge bands when using time series images can improve the crop classification accuracy. Adding the NDVI and EVI vegetation indices (experiment 3) fails to improve the classification accuracy of experiment 2 (the difference between the lowest and the greatest accuracies is 0.02%). When using the time series images for crop classification, experiment 5 obtained the best classification performance (95.02% and 0.93), while experiment 4 had the lowest classification (86.35% and 0.81).

**Figure 10:**
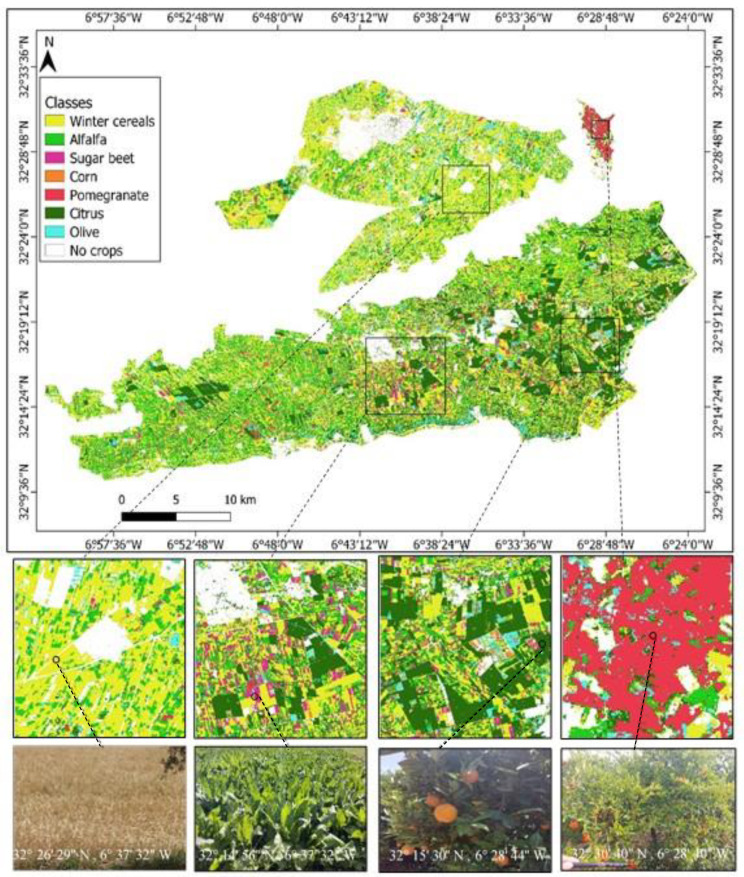
Crop classification result of Tadla Irrigated Perimeter based on Sentinel-1+ Sentinel-2 inputs and RF classifier in the early season.

As a result, experiment 5 was selected to map the crop distribution in the TIP area (Figure 10). Regarding the distributions of the main TIP crops, winter cereals and alfalfa cover a large area, particularly around the Beni Amir perimeter. Sugar beet is mostly found in the center of the Beni Moussa perimeter. Pomegranate trees are distributed in the northern sub-section of Beni Amir. Citrus and olive trees are located in the southeastern part of Beni Moussa, while corn is poorly distributed in the area.

### 5.4. Crop Mapping in the Early Season with Monthly Windows (Scenario 2)

The monthly window was chosen as the temporal window for evaluating the performance of TIP crop classification throughout the growing season. Table 5 reveals that the classification accuracies of experiment 2 and experiment 3 are greater than that of experiment 1 in all the months, which proves that adding SWIR bands, red-edge bands, and vegetation indices improves the classification accuracy when using single monthly window images.

Experiment 5 demonstrated consistently higher accuracy values for all months, demonstrating the importance of SAR imagery as a supplement to optical imagery in significantly improving crop classification accuracy in the early growing season.

**Figure 11:**
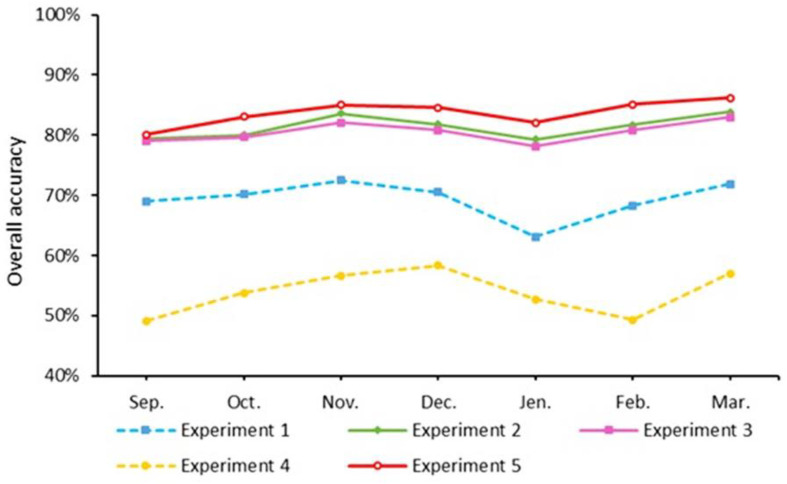
Classification accuracy as a function of experiments.

The classification accuracy of experiments varies over time; it increases from September to December, falls from December to February, and rises again from February to March. The highest classification accuracy and Kappa coefficient (86.22% and 0.81, respectively) were recorded for experiment 5 and the March monthly window. Experiment 2 and experiment 3 provide similar changes in accuracy and achieve an OA of more than 80% in most months (Figure 11).

### 5.5. Crop Area Forecasting in the Early Season

**Figure 12:**
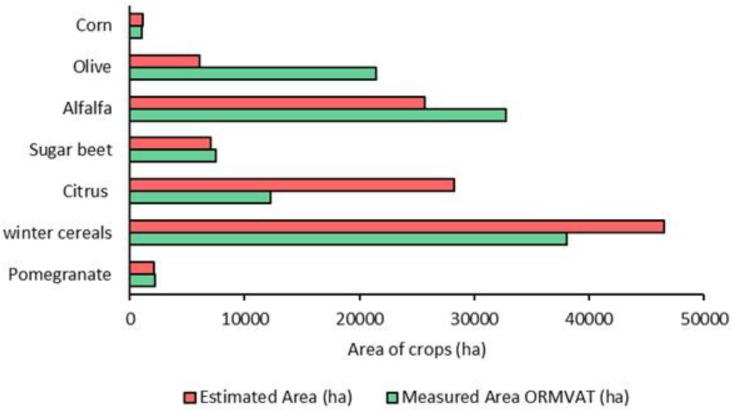
Estimated area using the RF classification and measured area based on the Tadla Agricultural Development Regional Office (ORMVAT) for the TIP crops.

**Figure 13:**
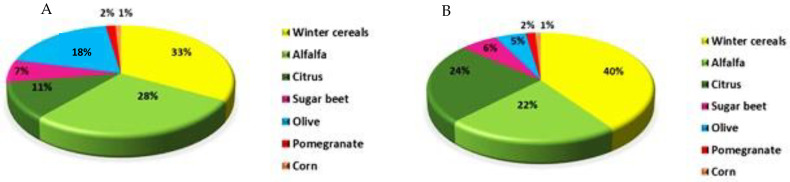
Spatial contribution of major crops in the Tadla Irrigated Perimeter for the year 2020/2021. (**A**) Measured contribution, (**B**) Estimated contribution.

In this section, the resulting image of classification from the fusion of optical and SAR data (experiment 5) with the time series from September to March (early season) was investigated to estimate the area of the TIP crops. The area of crops is calculated by multiplying the number of pixels classified into a class by the area of each pixel (Table 6). The comparison of crop areas obtained from the classification maps and the areas presented by ORMVAT (Figure 12) yields similar values for the majority of crops, particularly pomegranate, sugar beet, alfalfa, and winter cereals. However, differences in area values are recorded for olive and citrus. Figure 13 depicts the contribution of each crop class in Tadla’s irrigated area for the growing season 2020/2021 obtained from the area measured by the ORMVAT and the estimated area. Winter cereals and alfalfa contributed the most in the TIP region for the season 2020/2021.

## 6. Discussion

The open access remote-sensing datasets and the computing power of the GEE platform were key to achieving the objectives of this paper. This study included 38 Sentinel-2 Level 2-A data and 45 Sentinel-1 data, used to produce the median monthly composite images. These images were evaluated with five experiments and two scenarios to produce accurate crop classification results. Without the use of cloud-computing capacity, this amount is challenging to process. Furthermore, this could have taken several days, which delays decision-making in many cases. With GEE, on the other hand, this process was fast and required only a few seconds. However, most of the time was spent developing appropriate code to evaluate the various band reflectance values, vegetation indices, and backscattering coefficients on crop classification. Besides, two scenarios were used to evaluate the monthly temporal windows on classification accuracy.

Crop classification in a highly heterogeneous and fragmented agricultural region is challenging due to the temporal and spatial resolutions of images. On the other hand, mapping crops in early March is also a major challenge in this region. As a result, this study involved five combinations of parameters derived from optical and SAR imagery and two temporal scenarios to classify crops accurately. Results revealed that the highest accuracy of crop classification using the entire monthly series of images (OA = 95.02% and k = 0.93) is much higher than the greatest accuracy using single monthly images (OA = 86.22% and k = 0.81). This finding is consistent with previous studies, as LUO et al. [10] discovered that the classification performance using time series images is considerably better than using single-period images, and Inglada et al. [19] found that using SAR and optical image time series allows for the accurate generation of early crop-type mapping.

**Figure 14:**
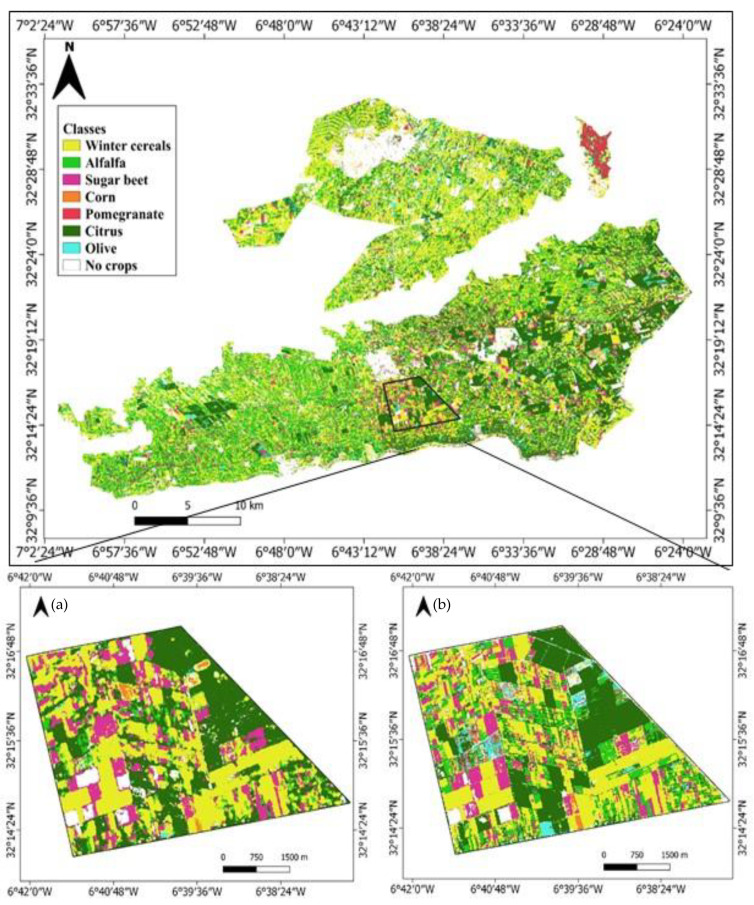
A detailed zoom for the crop classification results when using Sentinel-1 (**a**) and Sentinel-2 (**b**).

According to the results of experiments, adding red-edge and SWIR bands to the visible and NIR bands improves crop classification accuracy by 1.72%, while adding NDVI and EVI bands enhances crop classification accuracy by 0.02%. Consequently, Figure 14 reveals that when these bands are combined with the traditional bands, the accuracy of the TIP crops improves. For example, the accuracy of sugar beet is lower when only traditional bands are used for classification (F1 score = 86.87%), while adding SWIR and red-edge bands to the traditional band improve the classification accuracy (F1 score = 91.16%). This finding suggests that sugar beet can be detected using the red-edge and SWIR bands.

**Figure 15:**
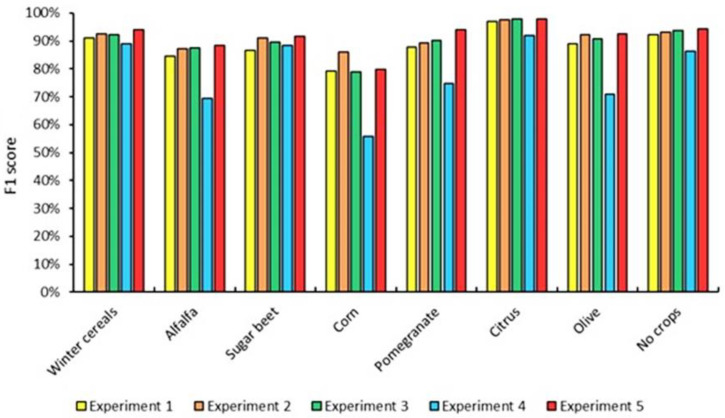
F1 score of TIP crops as a function of experiments.

However, the contribution of vegetation indices (NDVI, EVI) improves the accuracy of pomegranate classification (Figure 15).

The fusion of optical and SAR parameters yields the greatest accuracy (95.02% and 0.93). This accuracy is slightly higher than Sentinel-2 images alone (93.82% and 0.92), while it is considerably greater than when only Sentinel-1 images are used (86.35% and 0.81). These results indicate that the combination of optical and SAR data can accurately generate early crop mapping in a highly heterogeneous and fragmented area, as in the case of the TIP region. In India, Qadir and Mondal [42] found that the combination of SAR and optical data improves the monsoon cropland detection with an OA = 93%.

Figure 14 shows that the classification result using Sentinel-1 only in the large plots is not noticeably lower than Sentinel-2, whereas the areas characterized by small plots show confusion between classes when using only Sentinel-1 data.

The classification result obtained from the fusion of Sentinel-1 and Sentinel-2 features was evaluated by comparing the office and estimated area of crops. Results proved that the most accurate areas were assigned to pomegranate, sugar beet, alfalfa, and winter cereals, while differences in area values were recorded for olive and citrus (Figure 12). This gap can be related to the recently cultivated land characterised by small trees, which causes confusion between the two classes.

When compared to the results obtained by Ouzemou et al. [4] from pan-sharpened Landsat 8 NDVI data for the TIP crop mapping (OA = 89.26% and k = 0.85), the findings of this paper provide an accurate crop classification with more classes (OA = 95.02% and k = 0.93).

**Figure 16:**
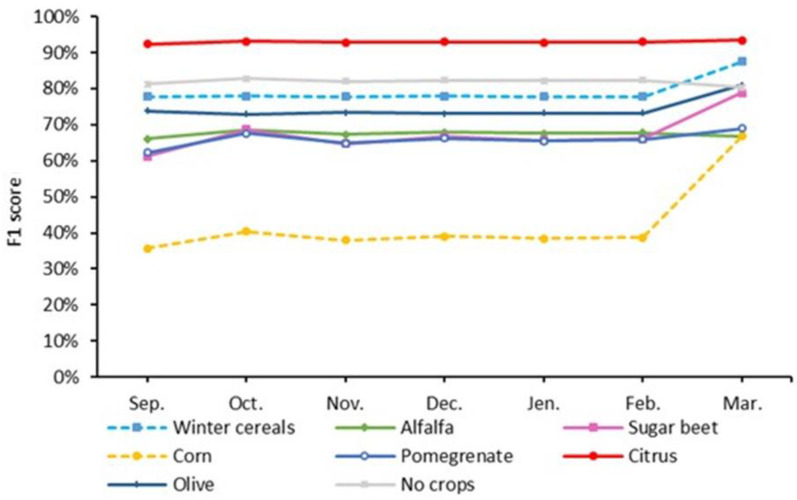
Variations in the F1 scores over time for experiment 5 (Sentinel-1 + Sentinel-2).

Early crop mapping could have several benefits, such as improved decision-making on agricultural practices, increased yields, and water resource management. Figure 16 shows that when using experiment 5, the accuracy of winter cereals, sugar beet, and corn all increase considerably in the early season, beginning in March.

This work proves that the fusion of Sentinel-1 and Sentinel-2 data can be used to identify crops in a highly heterogeneous and fragmented agricultural region throughout the early season as soon as the March images are available, with an OA of more than 80%.

## 7. Conclusions

In many different sorts of research, crop mapping from satellite images is extensively investigated using machine learning. However, very limited studies have investigated the efficiency of using Sentinel-1, Sentinel-2 data, and the RF algorithm on the GEE cloud computing platform. In this paper, GEE was used to apply multi-temporal Sentinel-1 and Sentinel-2 imagery for early crop mapping of a highly heterogeneous and fragmented agricultural region using an RF classifier under different scenarios and experiments. This study concludes that the fusion of all optical (Sentinel-2) and SAR (Sentinel-1) data (experiment 5) allows for an accurate crop mapping in the early season of growing, starting in March, for highly heterogeneous and fragmented landscapes, with an OA reaching 95.02%.

This research also suggests that the classification performance using monthly series images is considerably better than that using single-monthly images. Furthermore, the GEE processing was highly efficient at gaining access to remote sensing data and saving time.

Future work should focus on the use of GEE cloud computing and a multi-sensor harmonized image time series to improve crop classification accuracy in the earliest growing season of crops.

## Figures and Tables

**Table 2:** Number and area of selected ROIs.

Classes	Number of ROIs	Area (ha)
Winter cereals	229	336
Alfalfa	184	146
Sugar beet	78	123
Corn	22	195
Citrus	146	596
Pomegranate	27	248
Olive	32	113
No crops	18	979

**Table 3:** Date of acquisition of Sentinel-2 and Sentinel-1 from September 2020 to March 2021.

Month	Date of Acquisition Sentinel-2 (MSI)	Date of Acquisition Sentinel-1 (SAR)
September	3, 5, 10, 15, 20, 25, 28	2, 8, 14, 20, 26
October	5, 10, 13, 25, 28	2, 8, 14, 20, 26
November	2, 17, 22, 24	1, 7, 13, 19, 25
December	2, 12, 22, 27	1, 7, 13, 19, 25
January	1, 16, 26, 28	6, 12, 18, 24
February	2, 10, 15	5, 11, 17, 23
March	11, 22	6, 12, 18, 24
April	1, 13, 23	5, 11, 17, 23, 29
May	6, 11, 16	5, 11, 17, 23, 29
June	2, 12, 20	4, 10, 16

**Table 4:** Vegetation Indices and their expressions used in this study.

Index	Equation	S-2 Bands Used	Original Author
**NDVI**	(NIR − R)/(NIR + R)	(B8 − B4)/(B8 + B4)	[33]
**EVI**	2.5(NIR − R)/(NIR + 6R − 7.5 × BLUE + 1)	2.5(B8 − B4)/(B8 + 6B4 − 7.5 × B2 + 1)	[34]

**Table 5:** Classification accuracy of experiments based on monthly windows.

	Sept.		Oct.		Nov.		Dec.		Jen.		Feb.		Mar.	
	OA (%)	k	OA (%)	k	OA (%)	k	OA (%)	k	OA (%)	k	OA (%)	k	OA (%)	k
**Experiment 1**	68.95	0.57	70.14%	0.61	72.50	0.63	70.53	0.60	63.12	0.51	68.32	0.57	71.89	0.62
**Experiment 2**	79.37	0.72	80.01%	0.75	83.58	0.78	81.82	0.75	79.26	0.72	81.67	0.75	83.89	0.78
**Experiment 3**	79.01	0.71	79.67%	0.72	82.07	0.76	80.82	0.74	78.10	0.70	80.82	0.74	82.96	0.77
**Experiment 4**	49.10	0.28	53.80	0.34	56.64	0.37	58.32%	0.42	52.67	0.33	49.27	0.28	56.96	0.38
**Experiment 5**	80.07	0.73	83.06%	0.77	84.99	0.79	84.59%	0.79	82.05	0.75	85.14	0.78	86.22	0.81

**Table 6:** The number of pixels and estimated area based on the TIP crop classification result for the season 2020/2021.

Classes	Number of Pixels	Estimated Area (ha)
Pomegranate	209,388	2094
Winter cereals	4,656,382	46,564
Citrus	2,828,423	28,284
Sugar beet	706,455	7064
Alfalfa	2,566,989	25,670
Olive	609,967	6100
Corn	109,354	1093

## Data Availability

GEE is a free and open-source software program. All produced or utilized models, data, or codes available from the corresponding author during this study when requested.
